# The Bristol Royal Infirmary as a Trust Hospital—The First Six Months

**Published:** 1991-09

**Authors:** John Roylance, M. G. Wilson


### The Bristol Royal Infirmary as a Trust Hospital

### The First Six months

Interview with **John Roylance**

Dr. John Roylance gave me an hour of his time and talked freely about his thoughts and experience of the first six months of running the newly formed United Bristol Healthcare NHS Trust. This is the old Bristol and Weston Hospital Authority grouping minus Weston Health Services which have now also become a Trust. Though working in cooperation with the South Western Regional Health Authority he is accountable to the Secretary of State. He has the power to appoint new staff or to terminate appointments, he holds the budget. He acts with a Chairman and a board of Directors, 5 executive and 5 non-executive.

Under the new dispensation money is no longer given to hospitals directly through the Regional Health Authority, but indirectly as a result of contracts made between the purchasers — the Budget Holding Group Practices and the District Health Authorities (D.H.As.), who purchase services for patients of non-budget holding practices. These contracts have now all been concluded but there will be a year of stability before opportunities for change will arise. There are at present only two group practices holding budgets in the area — at Stockwood and at Nailsea, they have £5,000 p.a. to spend for elective treatments on each patient, sums over that amount e.g. for super expensive treatment will be met by the D.H.A. who will also meet costs of emergency treatment. The contracts are with the hospital not the individual doctors, they can be block contracts with departmental directors for a number of operations or consultations and they can also make variable contracts. In computing costs each hospital has to take into account all its capital assets and their depreciation, this includes the hospital site value. Hospitals centrally placed in large cities will thus be at a disadvantage compared to peripheral hospitals. In the case of the BRI the effect of this is not expected to be crucial. The ultimate effect of this may be to favour the removal of hospitals from commercial and industrial sites to less expensive peripheral areas where people live and prefer to be treated. This may eventually solve the problem of the overprovision of hospital services in Central London, the solution of which has been made difficult by the natural disinclination to abolish or remove Hospitals of historic national importance, and the powerful influences they can exert.

Payment of staff salaries and wages will be made by the Trust hospitals but these will remain on a level with those paid generally in the NHS. Merit Awards are still paid by the NHS. In theory salaries could be varied to remunerate remarkable talent or to penalise poor performance. In the former case Trusts could make payments to Academic Staff equivalent to Merit Awards which under the present system are strictly rationed. In the latter case it is very unlikely that a manager would take such a step as it would produce an immediate sympathy for the victim and such cases are rare. The fact that all hospital activity is now monitored and the results available on computer means that doctors are more than ever likely to wish for a high performance rating.

Every year the NHS has spent more in real terms on the NHS and for the current year the Bristol District Authority (recently combined with Southmead and Frenchay Health Authorities) has a budget of some £300 million for a population of 800,000 or £375 per head of population. It has never been and never will be possible to provide for everyone every new advance in medical therapy. It has to be rationed. The total sum available for the NHS has to be decided by the Chancellor of the Exchequer. Formerly the Department of Health and the Regional Health Authorities would decide on the priorities and the allocations. Under the new system the way the money is spent will be decided by the Budget holding GPs and the DHAs. These decisions will be much more sensitive to local opinion and need. As every hospital has to provide a tariff for its services and every purchaser has to decide where to purchase them, there will inevitably be competition between hospitals and some Districts and practices will purchase services outside their own area. Initially however the pattern of referrals will remain much as it was formerly. However, as doctors have to refer patients to where their contracts have been made and these are mostly block contracts the choices of the GP and of the patient will be less elastic than before. Later the differing costs of the services at different hospitals will lead to changes in referrals on the one hand and on the other the expensive hospitals will try to bring their costs down.

It is often said that the cost of the accounting and extra administrative staff will negate or outweigh the gains from increased efficiency. Actually the BRI is now spending less on administration than before because the use of computers reduces the need for staff. Without this technological revolution the new system would not be possible. But there is no escaping the new technology, it has come into every aspect of life and has to enter that of health provision too.

Dr. Roylance believes that the new system of Trust Hospitals and the separation of purchasers from providers is an advance and provides checks and balances that have never existed before but it will take 5 years or more before it can be fully and satisfactorily in operation. If the Labour party wins the next election and carries out its promise to reverse the changes he will regret it but, as a democrat and a civil servant acting on behalf of the elected government, he will regard it as his duty to comply.

M. G. WILSON

